# Simulated Firefighting Task Performance and Physiology Under Very Hot Conditions

**DOI:** 10.3389/fphys.2015.00322

**Published:** 2015-11-09

**Authors:** Brianna Larsen, Rod Snow, Michael Williams-Bell, Brad Aisbett

**Affiliations:** ^1^School of Exercise and Nutrition Sciences, Faculty of Health, Deakin UniversityMelbourne, VIC, Australia; ^2^Centre for Physical Activity and Nutrition Research, Faculty of Health, Deakin UniversityMelbourne, VIC, Australia; ^3^Faculty of Health Sciences, University of Ontario Institute of TechnologyOshawa, ON, Canada

**Keywords:** work output, heat, hydration, thermal stress, exertion

## Abstract

**Purpose:** To assess the impact of very hot (45°C) conditions on the performance of, and physiological responses to, a simulated firefighting manual-handling task compared to the same work in a temperate environment (18°C).

**Methods:** Ten male volunteer firefighters performed a 3-h protocol in both 18°C (CON) and 45°C (VH). Participants intermittently performed 12 × 1-min bouts of raking, 6 × 8-min bouts of low-intensity stepping, and 6 × 20-min rest periods. The area cleared during the raking task determined work performance. Core temperature, skin temperature, and heart rate were measured continuously. Participants also periodically rated their perceived exertion (RPE) and thermal sensation. Firefighters consumed water *ad libitum*. Urine specific gravity (USG) and changes in body mass determined hydration status.

**Results:** Firefighters raked 19% less debris during the VH condition. Core and skin temperature were 0.99 ± 0.20 and 5.45 ± 0.53°C higher, respectively, during the VH trial, and heart rate was 14–36 beats.min^−1^ higher in the VH trial. Firefighters consumed 2950 ± 1034 mL of water in the VH condition, compared to 1290 ± 525 in the CON trial. Sweat losses were higher in the VH (1886 ± 474 mL) compared to the CON trial (462 ± 392 mL), though both groups were hydrated upon protocol completion (USG < 1.020). Participants' average RPE was higher in the VH (15.6 ± 0.9) compared to the CON trial (12.6 ± 0.9). Similarly, the firefighers' thermal sensation scores were significantly higher in the VH (6.4 ± 0.5) compared to the CON trial (4.4 ± 0.4).

**Conclusions:** Despite the decreased work output and aggressive fluid replacement observed in the VH trial, firefighters' experienced increases in thermal stress, and exertion. Fire agencies should prioritize the health and safety of fire personnel in very hot temperatures, and consider the impact of reduced productivity on fire suppression efforts.

## Introduction

Performing physical work under very hot ambient conditions has been documented as dangerous, potentially even fatal, for wildland fire personnel (Cuddy and Ruby, 2011; Baldwin and Hales, 2012). Given future climate predictions, it is likely that firefighters will be exposed to such hazardous conditions on a more regular basis (Liu et al., 2010; Hanna et al., 2011; Coumou and Rahmstorf, 2012). For instance, the 2009 Black Saturday bushfires (in Victoria, Australia) were accompanied by temperatures of 46.4°C and extremely low humidity levels, and were preceded by a record-breaking heat wave of 3 days above 43°C (Teague et al., 2010). Even so, little policy exists in the fire industry around extreme heat and wildland firefighting practice in Australia. An operations bulletin was released by the Victorian Country Fire Authority (CFA) in 2012, which provides generalized guidelines around the management of heat stress in “extreme” weather conditions (e.g., rotate crews where possible, drink fluids at regular intervals; Country Fire Authority, 2012). However, the document reflects “common-sense” recommendations, rather than policy derived from a scientific evidence-base. Thus, rigorous research into the effects of high ambient heat on firefighters' and their work performance is warranted, as such research could underpin future heat policies for fire agencies.

There is a breadth of knowledge surrounding heat and exercise physiology (Cheuvront et al., 2010; Nybo et al., 2014). However, most research has focused on temperatures ranging from 30 to 40°C; fewer have explored the more “extreme” ambient conditions (e.g., 45°C) firefighters may be exposed to on the fireground. Select groups who have researched extremely hot ambient temperatures (e.g., 41.8–125°C) have done so over very short durations (≤15 min) (Duncan et al., 1979), have used modes of exercise far removed from firefighting work (e.g., cycling; Caldwell et al., 2012), or have not compared their findings to a more temperate control condition (Bennett et al., 1993; Walker et al., 2014). The small body of relevant research suggests that heart rate (Rowell et al., 1966; Wilson et al., 1975; Sköldström, 1987), perceived exertion (Sköldström, 1987), core temperature (Rowell et al., 1966; Wilson et al., 1975; Sköldström, 1987), skin temperature (Sköldström, 1987), sweat rate (Wilson et al., 1975), and fluid intake (Wilson et al., 1975) are all elevated when treadmill walking in extremely hot ambient conditions (40–50°C) when compared to temperate ambient environments (15–25.6°C). However, wildland firefighting work is also characterized by manual handling actions such as dig, rake, and drag (Phillips et al., 2012), and thus, treadmill walking may not serve as the best proxy when quantifying the effect of high heat on the performance and physiological responses during fire suppression work. In an urban structure firefighting protocol, ambient temperatures of up to 89°C significantly increased heart rate, tympanic temperature, and perceived exertion compared with performing the same fire drills under cool conditions (13°C) (Smith et al., 1997). Conversely, construction workers in the United Arab Emirates have been observed to maintain steady heart rate, tympanic temperature, fluid intake, and urine specific gravity (USG) values when working in temperatures ranging from 32.5 to 49°C (Bates and Schneider, 2008). However, work productivity was not monitored during these studies (Smith et al., 1997; Bates and Schneider, 2008), which prohibits understanding of the potential trade-off between physiological homeostasis and the maintenance of work performance in very hot ambient conditions.

The aim of the present study was to assess the impact of very hot (45°C) and dry conditions on the performance of, and physiological and subjective responses to, a wildland firefighting manual-handling task when compared to the same work in a temperate environment (18°C). It is extremely likely that, in concert with past research, significant increases in thermal stress (e.g., core and skin temperature) and exertion (e.g., heart rate) will be observed in the heat. However, quantifying the magnitude of these changes when performing intermittent, firefighting-specific work tasks is paramount when developing evidence-based health and safety policy. Further, no research to date has utilized a moderate duration, intermittent manual-handling protocol to investigate the performance changes that may occur during very hot conditions. Therefore, the true novelty of the study lies in understanding the impact of very hot ambient environments on simulated wildland firefighting work performance. Though research investigating manual-handling work performance in very hot temperatures is not yet available to support a firm hypothesis, the current authors predict that firefighters work performance will be reduced in the “very hot” condition. Acquiring information on the productivity of personnel in various ambient conditions may be vital for fire agencies in managing their human resources, and ensuring wildfires are controlled as efficiently and safely as possible.

## Materials and methods

### Participants

Ten healthy male volunteer wildland firefighters participated in the study. Participants provided written informed consent, and filled out a medical questionnaire to ensure they were physically able to perform the work protocol. Ethical approval was obtained from the Deakin University Human Ethics Committee. Participant's height was measured and recorded without shoes using a stadiometer (Fitness Assist, England). Semi-nude body mass (i.e., in underwear only) was measured using an electronic scale (Tanita, USA) pre- and post-exercise. In all trials, participants wore their own firefighting protective clothing, including a two-piece jacket and trouser set made from Proban® cotton fabric (Protex®, Australia), suspenders, boots, gloves, and helmet (amounting to ~5 kg). All testing took place during the winter months to limit heat acclimatization, which could have potentially confounded results.

### Experimental protocol

Participants were familiarized with the physical tasks, as well as the rating of perceived exertion (RPE) and thermal sensation scales, in a separate session within a week of testing (in 18°C) in order to minimize the chance of a learning effect (Hopkins et al., 2001). During the familiarization session, participants performed two sets of the 60-s rake bout (and thereafter provided practice RPE and thermal sensation ratings), and one 8-min step test (see *Raking task* and *Step test*). In the 24 h prior to testing, participants documented their activities (e.g., diet, sleep, and exercise behaviors), and were asked to replicate the same behaviors as closely as possible prior to both trials. Participants were instructed to abstain from alcohol and hard exercise, and to ensure they received adequate fluid intake and sleep, in order to minimize the risk of heat illness (Armstrong et al., 2007).

Participants ingested a core temperature capsule (Jonah, Minimitter, Oregon), a method of core temperature measurement that has been validated against both rectal and esophageal temperature (O'Brien et al., 1998), 6–8 h prior to testing. This allowed adequate time for the pill to pass through the stomach into the intestines to minimize inaccurate readings occurring as a result of ingested food or liquid (Lee et al., 2000). Core temperature results recorded on a data logger (worn in firefighters' jacket pocket) at 1 -min epochs throughout the testing period (VitalSense, Minimitter, Oregon). Firefighters were also instructed to slowly consume water (~5–7 mL.kg^−1^) in the 4 h prior to testing, to promote adequate hydration (Sawka et al., 2007). Upon arrival to the testing facility, participants had heart rate monitors (Polar, Finland), and skin temperature patches (VitalSense/Jonah, Minimitter, Oregon) affixed. Skin temperature was recorded at four sites on the left side of the body; the chest, thigh, upper arm, and calf (Payne et al., 1994).

Participants performed the protocol on two separate occasions, separated by at least 1 week to allow full recovery between trials. One session was conducted in a temperate environment (CON), and the other under very hot and dry conditions (VH). Trial order was counterbalanced. All testing was conducted in a climate chamber (Vötsch, Germany) which displayed ambient temperature and humidity readings (recorded at 10-min intervals). The climate chamber temperature was 18.0 ± 0.0°C in the CON trial and 45.0 ± 0.3°C in the VH trial (*P* < 0.001). Ambient humidity was 55.7 ± 1.2% in the CON trial, compared to 26.9 ± 2.0% in the VH condition (*P* < 0.001). A fan was used to provide a light breeze, to more realistically simulate an outdoor environment. Wind speed (measured at four sites in the chamber, and averaged) was maintained at <1 m.s^−1^ across both trials. Participants performed 3 h of intermittent, simulated rakehoe work (see *Raking task*) interspersed with a low-intensity stepping test (Siconolfi et al., 1985). A 3-h protocol was used as a compromise between simulating long-duration wildland firefighting work, and ensuring participants safety when performing physical work in very hot temperatures. Participants consumed water *ad libitum* throughout testing. Drinking water was maintained at 14.7 ± 0.6 and 15.2 ± 0.5°C in the CON and VH conditions, respectively (*P* = 0.074).

#### Raking task

The raking task simulated building a firebreak using a rakehoe. Rakehoe work was chosen due to its prevalence in different types of wildland firefighting (Phillips et al., 2011). Job task analysis research describes this task as short but intense, typically lasting 38–461 s on average (Budd et al., 1997; Phillips et al., 2011). The task simulation involved raking 29 kg of rubber tire crumb from one end of a rectangular (2 × 0.9 m) wooden box to the other repetitively, using a rakehoe. One “repetition” comprised participants raking the vast majority of the tire crumb from one half of the box (over a dividing line in the middle) into the other half. Participants had to wait until the researcher was satisfied that they had cleared enough material before progressing to the next end. The same researcher counted the repetitions for each participant, to ensure a consistent standard was being met. Rakehoe work performance was evaluated and compared between the CON and VH trials based on the number of repetitions participants were able to complete within the work periods (to the nearest quarter). Repetitions were converted to area (m^2^) for analysis.

#### Step test

The present research utilized a modified version of a sub-maximal step test (Siconolfi et al., 1985), to simulate the lighter intensity activity (e.g., periodic walking/hiking) performed on the fireground (Aisbett and Nichols, 2007; Raines et al., 2013). Only the lowest intensity phase of the test was utilized, as the energy expenditure of walking “with purpose” has been estimated at 4 METs (Powers and Howley, 2008). The test comprised repeatedly stepping up and down a 25-cm platform at a rate of 17 steps.min^−1^ (Siconolfi et al., 1985), as timed by a metronome. Participants who completed both trials were able to perform all of the prescribed stepping bouts in full. Thus, stepping performance was not included in the analysis.

#### Work to rest ratios

Over the course of a work shift, wildfire fighters have been observed to have periods of predominantly sedentary activity interspersed with brief spurts of moderate/vigorous activity (Cuddy et al., 2007; Raines et al., 2013). For example, mean time spent in the sedentary range for any given 2-h block of a 12-h workday has been observed to be 60.9–79.5 min.2 h^−1^ (Cuddy et al., 2007; Raines et al., 2013), which equates to spending 51–66% in the sedentary range. Further, 43.2 ± 24.2 min of any 2-h period is spent performing light intensity activity (Raines et al., 2013), with only 3.9–8.3 min.2 h^−1^ spent in the moderate/vigorous intensity range (Cuddy et al., 2007; Raines et al., 2013).

In order to simulate the varied-intensity, intermittent nature of wildland firefighting work (Aisbett and Nichols, 2007; Cuddy et al., 2007), the current protocol was broken up into three 1-h bouts (T1, T2, and T3). During each hour, participants spent 4 min intermittently performing the rakehoe task (4 × 1-min bouts), 16 min intermittently performing the stepping task (2 × 8-min bouts), and 40 min resting in the testing environment (2 × 20 min). These rest breaks equate to spending 67% in the sedentary range, which is close to the upper limit observed during fire suppression work (Cuddy et al., 2007; Raines et al., 2013). However, previous research investigating work intensity on the fireground was conducted in more mild ambient conditions (e.g., peak temperatures ranging from 18.6 to 33.9°C; Raines et al., 2013). It is reasonable to assume that rest periods could increase in hotter ambient temperatures. Similarly, the present study employed only 1-min raking bouts, as it is likely that rest breaks could be taken more frequently when performing this task under very hot environmental conditions.

Participants were allowed to remove their helmet and jacket during the 20-min rest periods, as is common during rest breaks on the fireground (Raines et al., 2012). Participants left the climate chamber only to go to the toilet, or if heat illness symptoms presented. Any time spent outside of the environmental condition was recorded.

### Physiological and subjective measurements

Core temperature, skin temperature, and heart rate were recorded continuously throughout testing. Mean skin temperature was calculated using the formula 0.3(*t*_chest_ + *t*_arm_) + 0.2(*t*_thigh_ + *t*_leg_) (Ramanathan, 1964). Fluid intake was recorded across the testing period. Urine was sampled pre-, during-, and post-exercise, and USG analyzed (using a portable refractometer; Atago, Japan), to approximate changes in hydration status. Pre- and post-body weight was recorded (and adjusted for ingested and expelled liquids) to determine changes in body mass (%) and to estimate sweat loss. Participants were also asked to provide RPE (on a 6–20 point scale; Borg, 1998) and thermal sensation (on a 0–8 point scale; Young et al., 1987) ratings after each rake bout.

### Statistical analysis

All statistical tests were carried out using the IBM Statistical Package for the Social Sciences (SPSS V.22.0.0, Champaign, Illinois). The distribution of the data was evaluated using Shapiro–Wilk tests. All data (with the exception of “time spent outside” the climate chamber and the total number of rake bouts completed) were normally distributed. The difference between conditions in total area raked, ambient temperature and humidity, and drinking water temperature was analyzed using *t*−tests. Repeated measures analysis of variance (ANOVA) were performed for all other normally distributed variables, with condition (CON or VH) and time as the two within-participant factors. Where the ANOVA revealed a significant interaction, simple effects analyses were used to detect at which point the significant difference occurred. The “time spent outside” and “bouts completed” data was not normally distributed, and this could not be corrected via transformation of the data. Thus, Wilcoxin-Signed Rank tests were used to assess the difference in these variables between conditions. These data are presented as median (inter-quartile range), whereas all other data are presented as mean ± SD. Significance was set at *p* < 0.05. For the data analyzed using *t*-tests, *t*−values were converted into effect sizes (*r*) using the method described by Field (2013). For the non-parametric data analyzed using Wilcoxin-Signed Rank test, effect sizes (*r*) were calculated by converting the *z*−score into an effect size estimate (Field, 2013). For both of these types of data, 0.1, 0.3, and 0.5 are considered small, medium, and large effect sizes (Field, 2013). For all normally distributed variables analyzed using ANOVA, partial eta-squared ($\eta_{p}^{2}$) effect sizes are presented (Lakens, 2013). When interpreting partial eta-squared results, 0.01, 0.06, and 0.14 are considered small, medium, and large effect sizes, respectively (Richardson, 2011).

## Results

Participant details are reported in Table 1. There was no difference between conditions (*P* = 0.357; *r* = 0.21) in the “time spent outside” data, with firefighters spending a median of 0 (2) and 2 (2) min outside the climate chamber (for toilet breaks) in the CON and VH trials, respectively.

**Table 1:** **Participant details**.

**N**	**10**
Age (years)	41 ± 17
Height (cm)	180.4 ± 9.0
Weight (kg)	89.4 ± 8.8
BMI	27.6 ± 3.1
Firefighting experience (years)	12 ± 12

### Work performance

**Figure 1:**
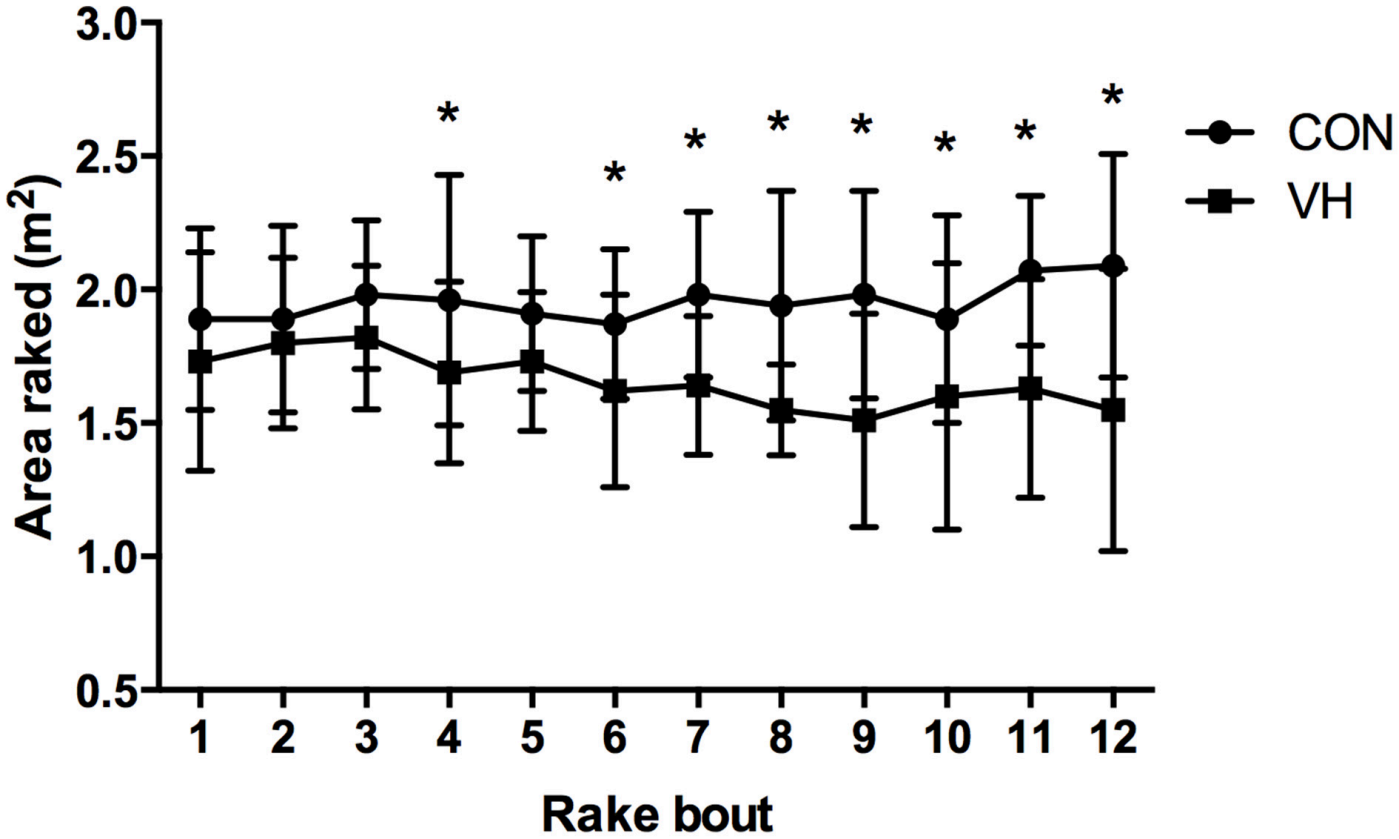
**Rake output (m^2^) during the 12 × 60-s rake bouts**. ^*^ Indicates that VH significantly lower (*P* < 0.05) than CON at individual time points.

All participants were able to complete the 3-h protocol in the CON trial, whereas two participants withdrew from the study due to heat illness symptoms in the VH condition after performing 9 and 10 (out of a possible 12) rake bouts, respectively. The difference in the number of bouts completed between conditions did not reach statistical significance (*P* = 0.180; *r* = 0.30). However, participants were able to clear 19% more total debris during the rakehoe task in the CON (23.45 ± 3.59 m^2^) compared to the VH trial (19.08 ± 4.24 m^2^; *P* < 0.001; *r* = 0.88). An interaction was also observed when the 12 × 60-s rake bouts were analyzed individually (*P* = 0.014; $\eta_{p}^{2}=0.11$), with firefighters raking significantly more in the CON compared to the VH condition during bout 4, and during all bouts from 6 onwards (*P* < 0.014; Figure 1).

### Core and skin temperature

Firefighters' baseline core and mean skin temperatures were not different between the CON (37.45 ± 0.31 and 31.09 ± 0.90°C) and VH (37.37 ± 0.18 and 31.59 ± 1.34°C) trials (*P* ≥ 0.240). There was, however, a significant interaction for hourly core temperature between conditions (*P* < 0.001; $\eta_{p}^{2}=0.62$). While there was no difference at T1 (*P* = 0.721), T2 and T3 reached 0.53 ± 0.18°C and 0.95 ± 0.17°C higher in the VH trial, respectively (*P* < 0.001; **Figure 3A**). Similarly, a significant interaction was observed for the peak core temperature reached each hour (*P* < 0.001; $\eta_{p}^{2}=0.50$). Again, the increase observed in the VH condition fell short of reaching significance during T1 (0.16 ± 0.21°C; *P* = 0.109), but was on average 0.63 ± 0.21 and 0.99 ± 0.20°C higher during T2 and T3 (*P* < 0.001; Table 2) when compared to the CON trial. There was no interaction between conditions for hourly mean skin temperature (*P* = 0.072; $\eta_{p}^{2}=0.11$), however there was a main effect observed for condition, such that firefighters mean skin temperature was on average 5.14 ± 0.48°C hotter across the VH compared to the CON trial (*P* < 0.001; $\eta_{p}^{2}=0.98$; **Figure 3B**). Conversely, a significant interaction was observed for the peak mean skin temperature reached each hour (*P* = 0.044; $\eta_{p}^{2}=0.13$). In this instance, the increase observed in the VH condition was significant at all time-points (*P* < 0.001), with participants reaching 4.57 ± 0.53, 5.07 ± 0.53, and 5.45 ± 0.53 higher during T1, T2, and T3 in the VH when compared to the CON trial (Table 2). Individual core temperature data was also plotted in Figure 2.

**Table 2:** **Peak core temperature, skin temperature, and heart rate over the 3-h work period**.

**Variable**	**T1**		**T2**		**T3**	
	**CON**	**VH**	**CON**	**VH**	**CON**	**VH**
Peak core temperature (°C)	37.71 ± 0.26	37.89 ± 0.21	37.73 ± 0.25	38.35 ± 0.21^*^	37.67 ± 0.21	38.65 ± 0.24^*^
Peak mean skin temperature (°C)	33.20 ± 1.05	37.77 ± 0.31^*^	32.98 ± 0.84	38.04 ± 0.43^*^	32.55 ± 0.80	38.12 ± 0.55^*^
Peak heart rate (beats.min^−1^)	148 ± 22	162 ± 21^#^	148 ± 21	168 ± 18^#^	147 ± 21	167 ± 18^#^

* *Indicates that VH significantly higher (P < 0.05) than CON at individual time points*.

# *Indicates VH higher than CON (main effect; P < 0.001)*.

**Figure 2:**
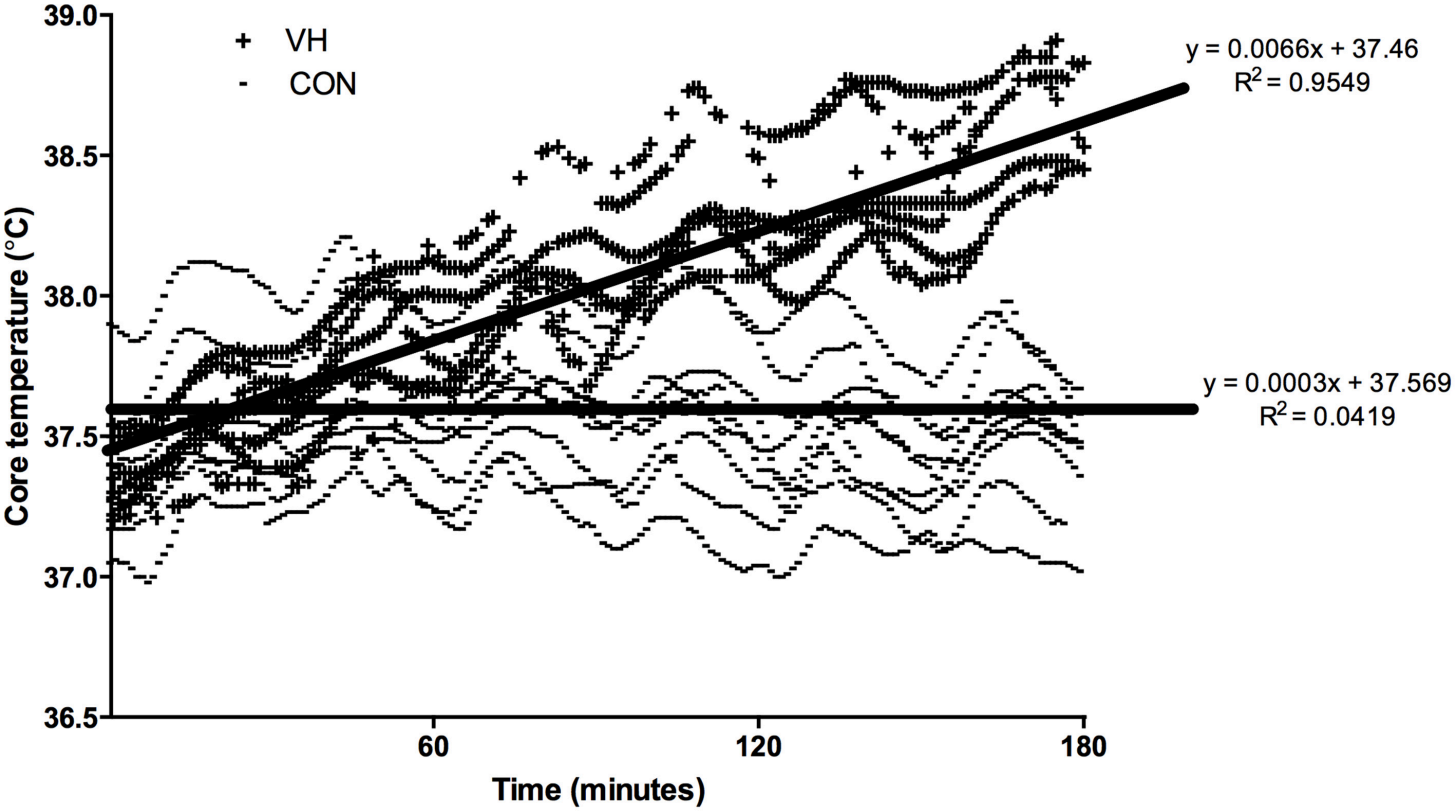
**Individual core temperatures over the 3-h work period**.

### Heart rate

**Figure 3:**
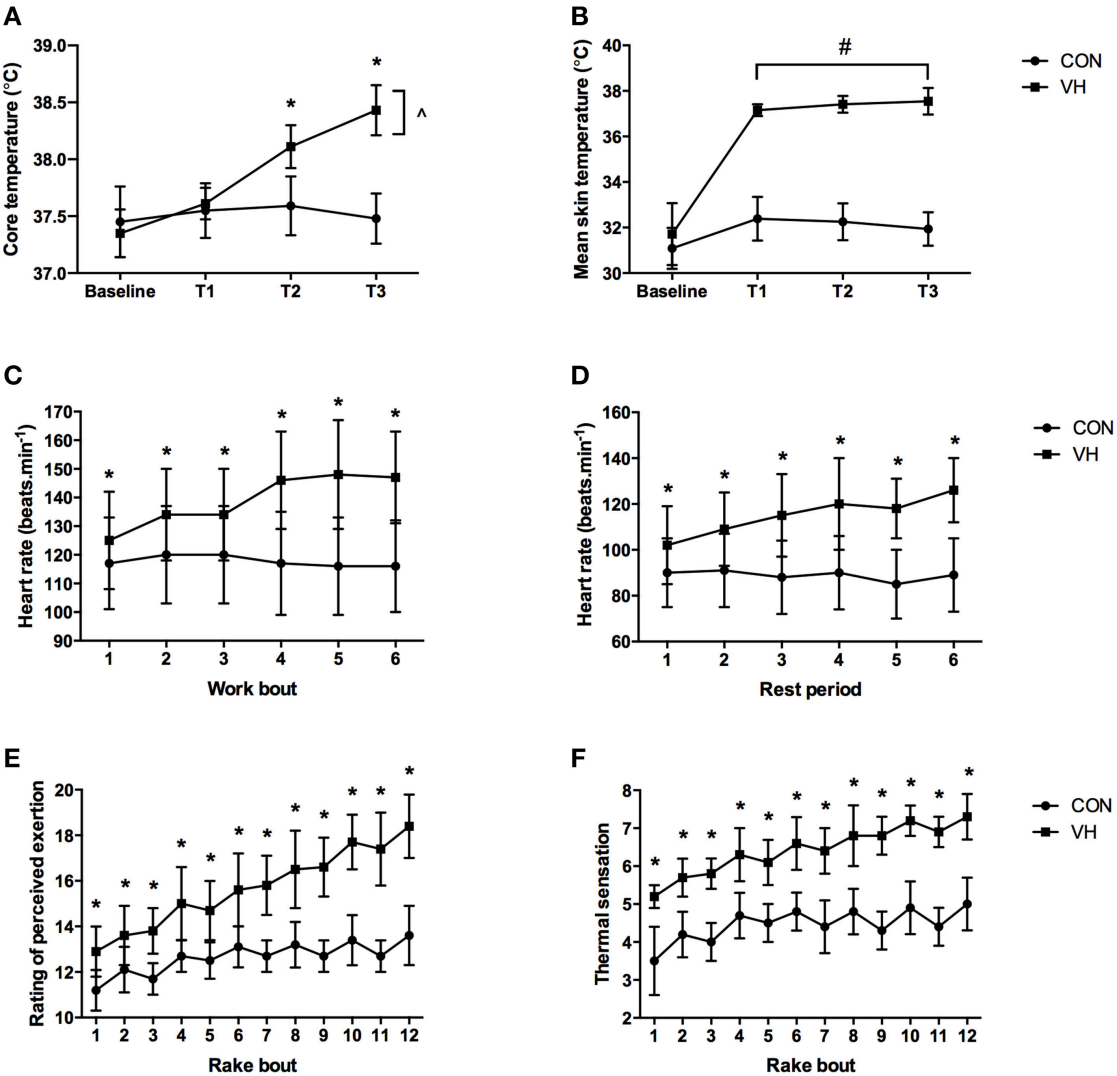
**Differences between the CON and VH conditions in: (A) hourly core temperature, (B) hourly mean skin temperature, (C) heart rate during the 10-min work bouts, (D) heart rate during the 20-min rest periods, (E) RPE after the 60-s rake bouts, and (F) thermal sensation after the 60-s rake bouts**. ^*^Indicates that VH significantly higher (*P* < 0.05) than CON at individual time points. ^∧^Indicates significant increase from T1 to T2, and T2 to T3 (*P* ≤ 0.004) in the VH. ^#^Indicates VH higher than CON (main effect; *P* < 0.001).

There was a significant interaction observed for mean hourly heart rate (*P* < 0.001; $\eta_{p}^{2}=0.41$), such that participants' heart rate was 100 ± 16, 98 ± 16, and 97 ± 15 beats.min^−1^ over T1, T2, and T3 during the CON trial, compared to 114 ± 16, 126 ± 18, and 133 ± 14 beats.min^−1^ in the VH condition (*P* < 0.001), respectively. Conversely, no interaction (*P* = 0.118; $\eta_{p}^{2}=0.09$) and no main effect for time (*P* = 0.271; $\eta_{p}^{2}=0.06$) were observed for firefighters' peak hourly heart rate. However, a main effect for condition highlighted that participants' peak heart rate was on average 19 ± 8 beats.min^−1^ higher across the VH compared to the CON trial (*P* < 0.001; $\eta_{p}^{2}=0.68$; Table 2). Heart rate data was also analyzed according to the periods of “work” (including both the rakehoe and stepping tasks) and rest. Time × condition interactions were observed for firefighters' heart rate during both the work and rest phases of the protocol (*P* < 0.001; $\eta_{p}^{2}=0.46$ and 0.33, respectively). Firefighters' heart rate was, on average, 22 ± 6 beats.min^−1^ and 27 ± 7 beats.min^−1^ higher in the VH compared to the CON trial for the periods of work (Figure 3C) and rest (Figure 3D), respectively.

### Perceptual responses

An interaction was observed for participants' RPE ratings (*P* < 0.001; η${}_{p}^{2}=0.27$), such that participants' RPE was significantly higher during the VH compared to the CON trial after each of the 12 rake bouts (*P* < 0.001; Figure 3E). The average RPE for the rake task in the VH condition was 15.6 ± 0.9 and categorized as “hard/heavy,” compared to 12.6 ± 0.9 (“somewhat hard”) in the CON trial. Similarly, an interaction was observed for participants' thermal sensation ratings (*P* < 0.001; η${}_{p}^{2}=0.16$). Again, this difference was statistically significant at each of the 12 time points (*P* < 0.001; Figure 3F). Firefighters felt hotter during the VH condition, rating their thermal sensation on average as 6.4 ± 0.5, compared to 4.4 ± 0.4 during the CON trial. The average thermal sensation in the VH trial signified “hot-very hot,” whereas average thermal sensation in the CON trial was “comfortable-warm.”

### Hydration

Firefighters consumed 2950 ± 1034 mL of water in the VH condition, compared to only 1290 ± 525 in the CON trial (*P* = 0.001). Conversely, there was no difference in urine output between conditions (*P* = 0.126), with firefighters producing 930 ± 783 and 634 ± 414 mL of urine in the CON and VH conditions, respectively. Firefighters in the VH condition did, however, have higher (*P* < 0.001) estimated sweat losses, reaching 1886 ± 474 mL compared to only 462 ± 392 mL when in the CON environment. There was no interaction (*P* = 0.506; $\eta_{p}^{2}=0.04$), and no main effects for condition (*P* = 0.170; $\eta_{p}^{2}=0.06$) or time (*P* = 0.269; $\eta_{p}^{2}=0.02$), observed for participants' USG scores pre-, during-, and post-work. Firefighters elicited pre-work USG scores of 1.014 ± 0.008 in both trials. During- and post-work USG scores reached 1.011 ± 0.005 and 1.016 ± 0.006 in the CON trial, compared to 1.017 ± 0.008 and 1.018 ± 1.007 in the VH condition. Thus, firefighters in both conditions were in the “hydrated” range (<1.020) at all time-points measured (Sawka et al., 2007). Further, there was no difference in the percentage body mass change between trials (*P* = 0.265). Participants in the CON trial lost 0.1 ± 0.9% of their body mass across the course of the protocol, whereas participants in the VH condition gained 0.5 ± 1.0%.

## Discussion

As predicted, firefighters' self-selected work output was reduced in the VH compared to the CON trial, which was reflected during both the individual rake bouts and the total amount of debris raked across the course of the protocol. Further, all measures of thermal stress (including core temperature, skin temperature, and thermal sensation) were elevated in the VH compared to the CON trial. Participants' heart rate and RPE were also significantly higher in the VH condition. However, firefighters' hydration status in the VH trial was not significantly different (in terms of their percentage body mass change and USG scores) than the CON trial, despite having higher sweat losses. This difference was offset by increased fluid intake in the VH environment.

To the current authors' knowledge, no previous research has evaluated the effects of very hot ambient temperatures (45°C) on self-paced, manual handling work performance (such as firefighting). Previous heat research investigating self-paced work (albeit usually employing different modes of exercise to firefighting) has typically observed one of two phenomena; either work output remains the same and physiological measures are elevated, or work output is decreased in an attempt to maintain thermal homeostasis (Cheung and Sleivert, 2004; Nybo et al., 2014). However, though participants in the current study performed 19% less work on the rakehoe task in the VH trial, significant increases across all measures of thermal strain (core temperature, skin temperature, thermal sensation) and exertion (heart rate, RPE) were recorded. It is possible, then, that the limits of self-pacing for modulating physiology were reached in the current protocol. Skin temperature, heart rate, RPE, and thermal sensation were significantly higher at all points during the VH trial, though participants remained hydrated throughout. Core temperature, on the other hand, was not significantly higher in the VH trial until T2 and T3. Given that the lower rake output was observed only in bout 4 and from bout 6 onwards, it is not unreasonable to assume that the increase in core temperature (in concert with the cardiovascular adjustments accompanying high skin temperatures) was the “trigger” behind firefighters self-selecting a lower work output relative to the CON trial. The interplay between increasing skin temperature, core temperature, and cardiovascular variables has been previously described as the primary explanation for impaired exercise performance in the heat (Cheuvront et al., 2010).

It must also be noted that, although not significant between conditions, two (of 10) participants in the VH trial withdrew before the end of the protocol due to experiencing heat illness symptoms (headaches and nausea). If, hypothetically, 20% of firefighters in the field were unable to complete their allocated shift length due to illness, this would have significant adverse follow-on consequences for the fire-suppression effort, as well as straining health support resources. This issue may be of particular concern for older firefighters, as they have been shown to have a reduced heat loss capacity relative to their younger counterparts (Kenny et al., 2015). However, unlike the current study, firefighters in the field would often be able to modify the length of their work bouts as well as their work intensity, which could further assist their ability to stave off heat illness symptoms. Nevertheless, the fire industry must consider the possibility of firefighter illness and “dropout” when operating under very hot fire weather conditions.

In addition to understanding the effect of very hot conditions on work performance, it is vital that the concurrent physiological changes are also quantified in order to develop policy that promotes and preserves the health and safety of personnel. Participants' core temperature was significantly increased in the VH compared to the CON trial, but perhaps not to the level that was expected based on the previous (albeit extremely limited) research in very hot temperatures (Rowell et al., 1966; Sköldström, 1987). Firefighters in the current protocol reached a peak core temperature of 38.65 ± 0.24°C in the third hour of testing. Conversely, Rowell et al. (1966) observed core temperatures of 39.4°C after 2 h of intermittent treadmill walking in 43.3°C, and Sköldström (1987) reported core temperatures of 38.7°C after just 1-h of low-intensity (3.5 km.h^−1^) treadmill walking in 45°C, while wearing PPC and breathing apparatus (BA). It is likely that the 1:2 work to rest ratio employed in the current protocol somewhat blunted the rise in core temperature, which may partially explain the differences when compared to the 1:1 and continuous work protocols utilized by Rowell et al. (1966) and Sköldström (1987), respectively. Firefighters also removed their helmet and opened their jacket during each of the 20-min rest periods, which would have helped reduce the thermal burden when compared to the fully-encapsulating PPC and BA utilized in the Sköldström (1987) study. Further, wind speed in these two studies was described as either “minimal” or <0.2 m.s^−1^; thus it is possible that the breeze provided by the fan in the present research (<1 m.s^−1^), in concert with participants' removal of helmets and opening of jackets, would have aided convective heat losses (Nybo et al., 2014).

The increased fluid intake observed in the VH trial also allowed for significantly higher estimated sweat losses, which in turn would have assisted in modulating core body temperature (Cheuvront et al., 2010). Indeed, firefighters managed to more than double their fluid intake in the VH condition, which also allowed them to maintain their body mass across the course of the trial. The USG findings also show that firefighters in both ambient conditions were classified as hydrated (<1.020) before, during, and after the work protocol. Firefighters in the field have also been observed to self-regulate fluid intake in order to complete their shift in a euhydrated state (Raines et al., 2012).

Despite the somewhat encouraging core temperature and hydration findings, participants' heart rate values were as much as 36 beats.min^−1^ higher in the VH when compared to the CON trial, despite performing significantly less work in the heat. Heart rate peaked at 168 ± 18 beats.min^−1^ in T2, which equated to 94 ± 12% of participants age predicted maximum heart rate (using the formula 207–0.7 × age) (Gellish et al., 2007). This is high given that the most intense task was performed in only 1-min bouts, and totaled only 4-min each hour. Recent research has shown that 37% of male volunteer firefighters in Victoria, Australia are considered at “high risk” of developing coronary heart disease in the next 10 years (Wolkow et al., 2013). Cardiovascular disease related fatalities are the leading cause of on-duty deaths in US firefighters, the majority of which occur in individuals with pre-existing risk factors (Wolkow et al., 2012). While regular physical activity may reduce the risk of mortality arising from cardiovascular events, vigorous exercise inducing high heart rates has been shown to increase the risk of acute cardiovascular events in untrained or at-risk populations (Dyer et al., 1980; Siscovick et al., 1984; Cobb and Weaver, 1986; Thompson et al., 2007). Thus, the high levels of physiological exertion experienced in the present study clearly illustrates that close monitoring of firefighter health is necessary and warranted during very hot wildfire conditions.

It is important to note that the current study may have some limitations that prevent direct extrapolation to the fireground. Firstly, the focus of this research was on ambient heat, whereas firefighters in the field would also be exposed to radiant heat from the sun, and in some cases, the fire. If anything, this means that the present findings may underestimate the thermal stress placed on personnel when on duty. Secondly, the wind speed utilized in the current protocol may not reflect the variations in wind speed/direction that would be experienced in an outdoor environment. Finally, though long in duration relative to past research in very hot temperatures, the 3-h protocol used may not serve as the perfect proxy for a firefighting shift. Although firefighters may not be exposed to ambient temperatures as high as 45°C for longer than a few hours, it is likely that they would perform physical work in hot conditions throughout the course of the day, and would begin their work in the “extreme” conditions (typically in the afternoon) with a higher starting core temperature and some level of physical or mental fatigue. While participants' core temperature in the VH condition perhaps rose more gradually than expected, it did not plateau; core temperature each hour was significantly hotter than the previous hour (Figure 3A). Thus, it is not unreasonable to assume that firefighters' performing a similar workload over a longer period would reach core temperatures likely to lead to heat exhaustion. For instance, if the trend line equation from Figure 2 was used to extrapolate the data, it is predicted that core temperatures could reach 39.84°C in the VH condition after 6 h of work. The wildland firefighting industry must continue to consider and evaluate strategies (e.g., shorter shift lengths, progressively longer rest periods between work bouts) in order to maximize the effectiveness of wildfire suppression efforts in very hot temperatures, while also preserving the health and safety of fire personnel.

## Conclusions

Firefighters in the present study recorded significantly lower performance values, and higher levels of thermal stress and exertion, in the 45°C condition. However, firefighters were able to self-regulate their water intake to prevent changes in body mass and USG (which serve as a proxy for hydration status). Further, the observed elevations in core temperature were relatively moderate when compared to previous research in similar ambient environments. It is likely that the frequent rest breaks employed in the current protocol aided in blunting the rise in core temperature, along with providing the firefighters with ample fluid replacement opportunities. However, given that core temperatures did not plateau over the course of the protocol, it is unlikely that the firefighters would have been able to continue at that rate of work over more extended periods. Fire agencies need to consider how they are going to manage the observed decline in work output in very hot conditions, in order to maximize the effectiveness of fire suppression operations and manage individual health and safety.

### Conflict of interest statement

The authors declare that the research was conducted in the absence of any commercial or financial relationships that could be construed as a potential conflict of interest.
